# The Effects of a Multiflavonoid Supplement on Vascular and Hemodynamic Parameters following Acute Exercise

**DOI:** 10.1155/2011/210798

**Published:** 2011-12-04

**Authors:** Rebecca M. Kappus, Chelsea D. Curry, Steve McAnulty, Janice Welsh, David Morris, David C. Nieman, Jeffrey Soukup, Scott R. Collier

**Affiliations:** Vascular Biology and Autonomic Studies Laboratory, Appalachian State University, Boone, NC 28608, USA

## Abstract

Antioxidants can decrease oxidative stress and combined with acute exercise they may lead to further decreases in blood pressure. The purpose of this study was to investigate the effects of 2 weeks of antioxidant supplementation on vascular distensibility and cardiovascular hemodynamics during postexercise hypotension. *Methods*. Twenty young subjects were randomized to placebo (*n* = 10) or antioxidant supplementation (*n* = 10) for two weeks. Antioxidant status, vascular distensibility, and hemodynamics were obtained before, immediately, and 30 minutes after an acute bout of aerobic exercise both before and after supplementation. *Results*. Two weeks of antioxidant supplementation resulted in a greater systolic blood pressure (SBP) decrease during postexercise hypotension (PEH) and significant decreases in augmentation index versus placebo (12.5% versus 3.5%, resp.). Also ferric-reducing ability of plasma (FRAP) increased significantly (interaction *P* = 0.024) after supplementation. *Conclusion*. Supplementation showed an additive effect on PEH associated with increased FRAP values and decreases in systolic blood pressure and augmentation index.

## 1. Introduction

Hypertension is defined as an elevation of blood pressure at or above a systolic blood pressure (SBP) of 140 mm Hg and/or a diastolic blood pressure (DBP) of 90 mm Hg. Pre-hypertension is designated as a systolic pressure from 120 to 139 mm Hg or a diastolic pressure from 80 to 89 mm Hg [1]. Chronic hypertension degrades the elastic properties of the arterial walls, leaving the vessel less compliant and unable to absorb the high energy of arterial outflow [2]. Healthy, large arteries contain elastin, smooth muscle, and collagen which allow them to be acquiescent and buffer the pressure change due to ventricular ejection [3]. This reduction in elasticity termed arterial stiffness leads to an increase in systolic blood pressure and a decrease in diastolic BP resulting in an increase in pulse pressure which has been shown to increase cardiovascular risk.

Hypertension is widespread in the western world and it imposes a substantial burden on health care costs in the United States. According to the TROPHY study, it is possible to delay the progression from prehypertension to hypertension with medication [4], however this strategy will expose tens of millions of individuals to patienthood and expensive pharmacologic treatments and unfavorable side effects that may be due to reactive metabolites [5]. Non-pharmacological treatments such as antioxidant treatments have been proven safe [6] and are in demand in order to ease the burden on costs and negative side effects associated with hypertension medications. However, there is little data showing evidence of one particular flavonoid having a significant effect on the aforementioned vascular parameters. Therefore, a compound of multiflavonoids may exude a more favorable effect on cardiovascular variables by increasing the bioavailability and absorption of the ingredients within the compound. Alternatively, it has been shown that there is a decrease in blood pressure called postexercise hypotension (PEH) following an acute bout of exercise which could potentially be an effective treatment for elevated blood pressure. PEH could be the first line of defense against hypertension in order to control blood pressure and therefore prevent disease. Additionally, antioxidant supplementation has been shown to decrease blood pressure by increasing endothelial nitric oxide synthase (eNOS) which will reduce acute exercise-induced inflammation and oxidative stress [7–10]. Flavonoids in particular are strong antioxidants due to their low redox potential and capacity to donate several electrons or hydrogen atoms and the most widespread of all flavonoids is quercetin. In addition, both epigallocatechin 3-gallate (EGCG) and fish oil have beneficial effects on cardiovascular health and have been shown to decrease systolic blood pressure and enhance arterial elasticity [11, 12].

If it is possible to further decrease blood pressure during PEH due to these antioxidants and omega-3s, supplementation may be more favorable than pharmacological anti-hypertensives. The primary focus of this study was to investigate the potential additive effects of a novel omega-3 plus multiflavonoid supplement combined with aerobic exercise on PEH in young pre-hypertensives. We hypothesized that the supplement in combination with aerobic exercise will show an additive decrease in postexercise blood pressure (BP) and pulse wave velocity (PWV) changes while increasing arterial distensibility.

## 2. Materials and Methods

### 2.1. Participants

Prior to the start of this study, all methods were reviewed and approved by the University Institutional Review Board. Twenty participants (18 male, 2 female), 18–26 years old, were recruited through posters/fliers located throughout the University Community. Participants on any medication, including aspirin or birth control, and/or with known cardiovascular disease, diabetes, or hypertension were excluded. Prior to the start of the study, each subject gave written informed consent and completed health history and lipid profile questionnaire to assess prior activity level [13]. The sample size of 10 subjects per group was based on previous data from our laboratory gathered under similar conditions. For these calculations, the STATA statistical software package was used (College Station, TX) to determine the number of subjects to give us adequate statistical power. We based our calculations on two dependent variables (MAP and Aix) and found their power to be 0.92 and 0.90, respectively, for the present sample size at a *P* ⩽ 0.05.

### 2.2. Study Design

Participants reported to a temperature controlled laboratory (22.5–23.5 degrees Celsius) on four separate occasions. Visit 1 consisted of informed consent, group randomization, body composition assessment (DEXA scan), and a laboratory equipment familiarization including laboratory equipment data collection procedures. This was followed by a graded exercise to test volitional exhaustion performed on a treadmill to determine VO_2peak_.

 The second visit consisted of a 12-hour fasted blood draw taken at 0700 hours by a certified phlebotomist in the laboratory. A 20 mL blood draw was obtained with the subject in the seated position using the antecubital vein. The blood was immediately centrifuged and the resulting plasma was aliquoted into cryovials and frozen in liquid nitrogen for later analyses of ferric-reducing ability of plasma and the oxygen radical absorbance capacity (FRAP, ORAC, resp.) and hematocrit.

Upon entering the laboratory for Visit 3, the subject was seated and rested quietly for 15 minutes and had a resting blood pressure taken in their left arm using a manual sphygmomanometer and a stethoscope using the first and fourth Korotkoff sounds for SBP and DBP, followed by pulse wave analysis (augmentation index, AIx) measurements in the radial artery. Next, the subject was placed in a supine position and rested quietly for 10 minutes. Pulse wave velocity measurements were taken and then the subject was left in a quiet, darkened room while beat to beat blood pressure measurements were recorded for 10 minutes. Following these cardiovascular measures, the participants underwent a preexercise blood draw before running on the treadmill at a speed that elicited 65–70% of  VO_2peak_ HR for 30 minutes. Immediately following the termination of the exercise session, each participant underwent a seated postexercise blood draw and blood pressure measurement. Immediately following the blood draw and 30 minutes after exercise, each participant had the same measurements taken (AIx, pulse wave velocity and beat to beat measurements) immediately after and then 30 minutes following exercise.

After the third visit, the participants started a 14-day supplementation of an antioxidant mixture of quercetin, isoquercetin, omega 3 fatty acids, and EGCG, or a placebo. Both supplements were similar in composition and taste to a candied fruit chew, however the placebo lacked the active ingredients. The antioxidant daily dosage consisted of 1000 mg quercetin with 120 mg EGCG, 400 mg isoquercetin, and 400 mg omega 3 fatty acids from fish oil. The participants were instructed to take four chews a day of the supplement, with two taken immediately prior to lunch and two chews ingested immediately prior to dinner. All subjects were asked to maintain their daily eating habits and to return the empty packets of supplements at their next laboratory visit. Once the fourteen-day supplementation was completed, the participants reported back to the lab within 24 hours for the fourth visit and underwent the battery of tests performed in visit 3.

Due to the confounding nature of estrogen on our cardiovascular parameters, the female participants had to arrange their supplementation and visits around their menstrual cycle and one female was counterbalanced for each treatment. Both visits two and three were performed on day two of their menstrual cycle, and supplementation commenced two weeks later; therefore when the fourteenth day of supplementation was finished, the female participants were on day two of their cycle for visit four.

## 3. Description of Procedures

### 3.1. DEXA Scan

“Dual Energy X-ray Absorptiometry” or DEXA is a commonly used test for measuring bone mineral density and one of the most accurate ways to determine body composition. Subjects were placed in a Hologic Discovery QDR scanner in the supine position with their legs rotated inward to 25 degrees and a whole body scan was performed. Each scan was downloaded and analyzed by the same trained technician in one session.

### 3.2. VO_2peak_ Exercise Testing

Aerobic exercise capacity was assessed using a modified Armstrong protocol, briefly, the subject begins the test at a comfortable running pace (between 5.0 and 6.0 miles per hour) at 0% grade with the incline increasing 2.5% every two minutes until volitional fatigue was reached. If the subject did not reach fatigue when the grade reached 10%, the speed was then increased by 2 mph every 2 minutes. Heart rate was measured using a Polar Heart Rate Monitor (Polar Electro, Inc., Woodbury, NY, USA) and was recorded once during the final 15 seconds of every stage, along with a rating of perceived exertion RPE (Borg, 6–20). Expired air was collected continuously throughout the test and analyzed using a Quark b^2^ breath-by-breath metabolic system (Cosmed, Rome, Italy) for oxygen consumption, CO_2_ production, and respiratory exchange ratio. VO_2peak_ was determined by averaging the VO_2_ values over the final 15 seconds of exercise. Maximal effort was considered to have been reached when participants met three of the following four criteria: (a) an RER of 1.15 or greater; (b) a plateau in HR despite an increase in workload; (c) a final RPE score of 17 or greater on the Borg scale (scale 6–20); and/or (d) a plateau in oxygen uptake despite an increase in workload.

### 3.3. Pulse Wave Velocity (PWV)

All measurements were conducted in accordance with guidelines set forth by the Clinical Application of Arterial Stiffness, Task Force III [14]. A SphygmoCor was used to obtain the pulse wave between (1) the left common carotid artery and the left femoral artery and (2) the left common carotid and the left dorsalis pedis artery. Distance from the carotid sampling site to the midpoint of the manubrium sterni, manubrium sternum to femoral artery, and carotid to the midpoint of the manubrium sterni, manubrium sternum to dorsalis pedis was measured between these points as straight lines with a tape measure. The distance from the carotid artery to the manubrium sterni was subtracted from the manubrium to femoral artery distance. PWV was determined from the foot-to-foot flow wave velocity. The foot of the pressure wave was identified visually as the point of systolic upstroke. The time delay between a minimum of 15 simultaneously recorded flow waves was averaged. PWV will then be calculated from the distances between measurement points and the measured time delay (*Dt*) between proximal and distal foot waveforms as follows: PWV = *D*/*Dt* (m/s), where *D* is distance in meters and *Dt* is the time interval in seconds. Values attained from carotid to femoral artery were taken as an index of central compliance, while values attained from the carotid and radial artery along with the measurement from the femoral to dorsalis pedis were taken as an index of peripheral compliance. All data was stored and analyzed off line after completion of testing.

### 3.4. Hemodynamic Monitoring

Beat-to-beat blood pressures were determined via the Finometer (FMS, Amsterdam, The Netherlands). With participants in a supine position in a quiet and darkened room, beat-to-beat blood pressures were recorded for 10 minutes via finger plethysmography. This noninvasive measurement of the change in blood pressure has been shown to be reliable when compared with measurements of intra-arterial blood pressure [15]. The brachial blood pressure was obtained using an integrated brachial blood pressure cuff and brachial BP waveforms were reconstructed from finger arterial waveforms by applying an inverse transfer function, a waveform filter, a level correction, and a level calibration [16]. This has been shown to increase the correlation between finger and proximal arterial blood pressure values, allowing pressure values to remain within the American Association for Medical Instrumentation (AAMI) standards for the evaluation of automated sphygmomanometers [17].

### 3.5. Analyses: Biomarkers of Oxidative Stress

Fasted blood samples were drawn from an antecubital vein with participants in a seated position. The blood samples were centrifuged in EDTA tubes, and plasma was aliquoted and stored at 80°C prior to analysis for plasma antioxidant parameters Ferric Reducing Ability of Plasma (FRAP) and Oxygen Radical Absorptive Capacity (ORAC).

#### 3.5.1. FRAP

Total plasma antioxidant potential was determined by the FRAP assay according to the methodology of Benzie and Strain [18]. The basis of this assay is that water soluble reducing agents (antioxidants) in the plasma will reduce ferric ions to ferrous ions, which then react with an added chromogen. Samples and standards were analyzed in duplicate and expressed as ascorbate equivalents based on an ascorbate standard curve (0–1000 *μ*mol). Intra-assay and interassay coefficients of variation were less than 5% and 7%, respectively.

#### 3.5.2. ORAC

The ORAC assay was a modification of the methodology of Ou et al. [19]. Trolox standards were made from a trolox stock solution. A serial dilution of a 50 *μ*M trolox solution was made with phosphate buffer solution to produce 25, 12.5, and 6.25 *μ*M trolox standards. A fluorescein (Aldrich Chemicals) working solution was made by pipetting 800 *μ*L of stock solution into 50 mL phosphate buffer in a 50 mL conical tube. Before use, the solution was incubated in the water bath at 37°C until thoroughly heated. The 2,2′-azobis-2-methyl-propanimidamide, dihydrochloride (AAPH) solution was made by dissolving 0.108 g of AAPH (Wako Chemical) into 5 mL of incubated phosphate buffer immediately before the start of the assay. The microplate was prepared and loaded in a “forward-then-reverse” order to avoid possible positional errors. The edge wells were left empty or blank (phosphate buffer working solution) to reduce the impact of “edge effect” on samples and standards particularly from temperature effects on the outside wells. Twenty *μ*L of sample, blank, and trolox standard solutions were pipetted into appropriate wells. Then, 200 *μ*L fluorescein working solution were added to each well using an 8-channel micropipettor. A cover was placed on the microplate and the plate and contents incubated at 37°C for at least 20 minutes. Then, 20 *μ*L AAPH working solution were added using an 8 channel micropipettor as quickly as possible. Final ORAC values (*μ*M/L trolox) were made from the plate reader derived area under the curve (AUC). Then, the final ORACFL values were calculated by using a quadratic regression equation *x* = −*b* ± √*b* ^2^ − 4*ac* + 4*cy*. Excitation wavelength was 485 nm and emission wavelength was 520 nm. Intra-assay and inter-assay coefficients of variation were less than 5% and 7%, respectively.

### 3.6. Statistical Analysis

Student's *t*-test for unpaired values was used to evaluate the statistical differences in descriptive characteristics between groups. An ANOVA with repeated measures (group (treatment versus placebo) by time (baseline pre versus post) by exercise treatment (before versus after versus after 30)) was performed on all dependent variables. If a significant interaction was found, then the significant variables were compared using a Bonferroni post hoc test. SPSS version 17 (Chicago, Ill) statistical analysis software was used to perform all statistical calculations and all data is presented as means ± SEM.

## 4. Results

No significant differences between the antioxidant treatment group (*n* = 10) and the placebo group (*n* = 10) were found in age, height, weight, body mass, VO_2peak_, or resting heart rate which are presented in Table 1. There were also no significant differences between the treatment and placebo group in PWV (central or peripheral), resting SBP, DBP, heart rate (HR), or ORAC between groups.

**Figure 1:**
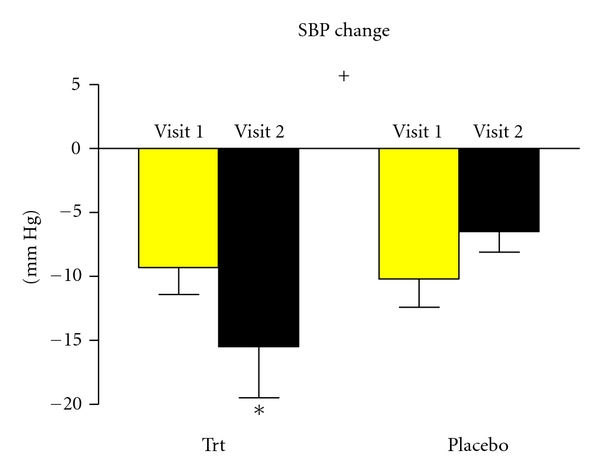
The change in systolic blood pressure from before and after acute exercise for visit 1 (presupplementation/placebo) and visit 2 (postsupplementation/placebo) amongst the trt group (*n* = 10) and the placebo group (*n* = 10). There was a significant decrease in systolic blood pressure following supplementation in the treatment group after an acute bout of exercise. ^+^denotes interaction (*P* = 0.04), *denotes significance (*P* < 0.05). Values are means ± SEM.

Significant differences were found in 30-minute postexercise systolic blood pressure measurements in the treatment group following supplementation (Figure 1). The placebo group had no significant SBP changes from before to after exercise following two weeks of placebo supplementation. The treatment group, however, had a further significant (*P* < 0.05) decrease in blood pressure following an acute bout of exercise after antioxidant supplementation.

**Figure 2:**
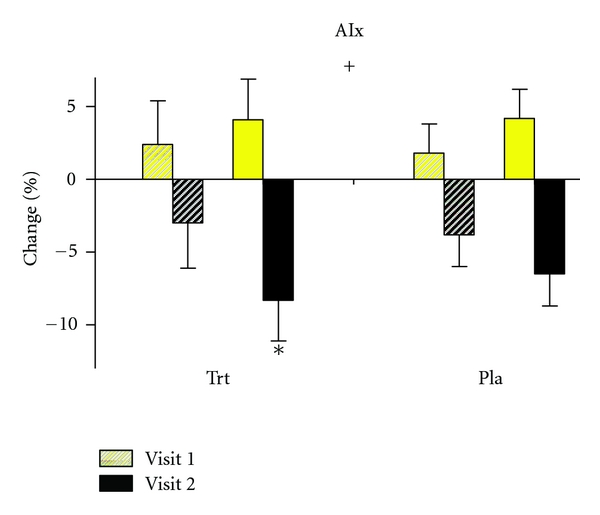
Augmentation index before and after acute exercise for visit 1 (presupplementation/placebo) and visit 2 (postsupplementation/placebo) for Trt group (*n* = 10) and placebo group (*n* = 10). There was a significant decrease in augmentation index in the treatment group following an acute bout of exercise after two weeks of supplementation. *denotes a significant change from before to after exercise (*P* = 0.01), ^+^denotes a significant interaction (*P* = 0.03) Values are means ± SEM.

As shown in Figure 2, AIx also showed a significant reduction in the treatment group following supplementation. After antioxidant supplementation, AIx showed a significant (*P* = 0.01) decrease from before to after exercise, whereas there were no significant changes in the placebo group and no significant changes before supplementation.

**Figure 3:**
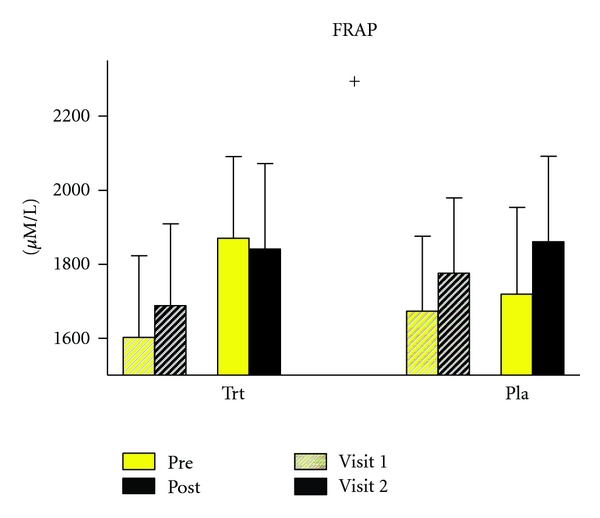
FRAP assay results before and after acute exercise for visit 1 (presupplementation/placebo) and visit 2 (postsupplementation/placebo) amongst the trt group (*n* = 10) and the placebo group (*n* = 10). There was a significant increase in resting FRAP levels from before to after supplementation in the treatment group only. *denotes significance from visit 1 to visit 2 (*P* = 0.001). Values are means ± SEM.

A significant (*P* = 0.001) increase was found in FRAP in the antioxidant supplementation group in the before exercise measurements. There were no significant changes in the placebo group following placebo supplementation. Following exercise, there were no significant differences in FRAP levels in supplement or placebo groups (Figure 3).

## 5. Discussion

Results from the present investigation support the theory that ingesting a multiflavonoid plus fish oil supplement in combination with aerobic exercise significantly reduces SBP and AIx at 30 minutes after exercise and significantly increases resting levels of FRAP in postsupplementation levels compared with presupplementation.

The attenuation of AIx, a measure linked to aortic arterial stiffness, was likely due to the significant increase in FRAP which may have led to greater bioavailability of NO within the elastic modulus of the aortic vessel. Since the amount of reflected wave was decreased, the amount of elastic “cushion” had to be augmented to absorb the pulsatile bolus of blood being ejected from the left ventricle. The increased expression of NO following flavonoid and fish oil supplementation has been shown in many studies [7, 10]. Interestingly, despite the decrease in AIx, there was no decrease in PWV as both are considered to be indicators or measures of arterial stiffness. This finding is not surprising since it has been shown on several occasions that AIx is not proportional to PWV in systolic hypertension, but instead has been shown to be inversely related [20]. In one study by Vyas et al. [21], AIx was inversely related to aortic PWV and weakly related to aortic compliance and an increase in AIx is not a reliable replacement for increased aortic stiffness. However, it did show that higher stiffness (increased PWV and lower compliance) was associated with a lower AIx [21]. The increased distensibility that resulted in a more favorable augmentation index could be due to the composition of the matrix of the vessel. The aorta has been shown to have the largest amount of eNOS potential and the supplement may have the greatest potential to upregulate this synthase pool.

Although the antioxidant supplementation did not have any effect on vascular distensibility (PWV) or resting SBP, the magnitude of PEH was significantly decreased in the 30-minute postexercise period after supplementation for the treatment condition when compared to the placebo without showing a correlating decrease in HR or PWV. Although resting SBP did not change with supplementation, there was a significant decrease in the systolic component of PEH. Because there was no change in PWV, one can only postulate that arterial distensibility was not the cause of this reduction in blood pressure. One possible explanation is that supplementation caused a decrease in oxidative stress, potentially due to an increase in antioxidant power (an increase in FRAP). A reduction in ROS would increase the bioavailability of NO, stimulating vasodilation. A second theory would be a change in sympathovagal regulation in which the supplement may induce a sympatholytic effect on sympathetic tone, thereby increasing the vagal tone.

The results of the present investigation support research done by Edwards et al. [22] who demonstrated a reduction in resting blood pressure in hypertensive versus pre-hypertensive patients following an antioxidant supplementation regimen of quercetin. Many studies have shown a beneficial decrease in resting blood pressure in hypertensives, indicating the possibility that this decrease in blood pressure can affect the hypertensive patient but not a normotensive or pre-hypertensive patient. To those authors' knowledge, this is the first investigation to support the premise that prehypertensives may also benefit from an antioxidant and n-3 fatty acid supplement. This also supports research demonstrating that antioxidants (like quercetin and Vitamin C) increased plasma quercetin levels but had no influence on oxidative stress or antioxidant capacity measures [23] and that chronic quercetin ingestion does not protect against exercise-induced oxidative stress or inflammation [24]. Without this protective mechanism against oxidative stress, it is possible that NO was not increased. Much of the research concerning antioxidant use has been shown to have beneficial effects in animal models [25–32], but there is a lack of benefits shown in human models. Speculatively, this could be due to the dosages ingested by humans being too large to be absorbed and simply excreted.

The significant increase in FRAP in the treatment group demonstrates that the total plasma antioxidant capacity increased following supplementation which may be related to the further decrease in systolic blood pressure during the postexercise period. This increase in antioxidant capacity could lead to an increase of free radical scavenging by antioxidants, thereby decreasing oxidative stress and potentially increasing NO levels. An increase of NO in the vasculature would lead to vasodilation and a further decrease of the systolic blood pressure after exercise. Interestingly, resting blood pressure following two weeks of supplementation was unchanged, demonstrating that a decrease in blood pressure in the 30-minute postexercise period from antioxidant supplementation occurred in conjunction with postexercise vasodilation.

The favorable effects of the supplement could be due to the multiflavonoid plus fish oil approach, unlike the single antioxidant approach that many previous research investigations have experimented with. The ingestion of many of the elements in the supplement has not shown consistent vascular benefits in humans. Chronic quercetin ingestion has not been proven to protect against exercise-induced oxidative stress or inflammation [24]. EGCG was shown to decrease stroke and mortality in stroke-prone, spontaneously hypertensive rats [11]. In relation to blood pressure, EGCG has been shown to decrease systolic blood pressure and enhances endothelial function and insulin sensitivity of spontaneous hypertensive rats, yet there is a paucity of the literature regarding the cardiovascular benefits of EGCG in humans.

People with a high risk of ischemic heart disease and/or hypertension could benefit from eating fish as clinical trials have shown that undergoing supplementation of fatty acids like fish oil can improve arterial elasticity in subjects with diabetes or dyslipidemia and even improved large arterial elasticity in overweight hypertensive patients [12]. A combination of fish oil and aerobic exercise has been shown to be more effective than individual components in decreasing triglycerides, increasing high-density lipoprotein (HDL), and improving endothelium dependent arterial vasodilation [33]. However, results from fish oil studies are equivocal. A 12-week double-blind crossover designed study by Lofgren et al. [34] found no change from pre-supplementation in systolic or diastolic blood pressure in middle-aged normotensive men [34].

In conclusion, we have found that a novel multiflavonoid plus fish oil supplement decreases augmentation index and systolic blood pressure with concomitant increases in FRAP, suggesting that cardiovascular benefits may be realized from this course of supplementation.

## Figures and Tables

**Table 1:** Participant characteristics.

Variable	Treatment	Placebo
*n*	10	10
Age (yr)	21.6 ± 0.62	20.7 ± 0.30
Height (cm)	177.4 ± 2.08	177.3 ± 2.57
Body mass (kg)	76.05 ± 3.24	75.55 ± 6.05
Peak oxygen consumption (mL/kg/min)	53.9 ± 3.88	47.9 ± 3.01
